# Epigenetic modification of miR-217 promotes intervertebral disc degeneration by targeting the FBXO21-ERK signalling pathway

**DOI:** 10.1186/s13075-022-02949-w

**Published:** 2022-11-28

**Authors:** Zhonghui Chen, Jianghua Ming, Yajing Liu, Geliang Hu, Qi Liao

**Affiliations:** 1 Orthopaedic Surgery, Fuzhou Second Hospital, Fuzhou, China; 2 The Third Clinical Medical College, Fujian Medical University, Fuzhou, China; 3 Orthopaedic Surgery, Renmin Hospital of Wuhan University, No. 9 ZhangZhiDong Street, Wuchang District, Wuhan, Hubei China; 4 Department of Anaesthesiology, Renmin Hospital of Wuhan University, Wuhan, China

**Keywords:** Intervertebral disc degeneration, miR-217, Methylation modification, FBXO21/ERK, Extracellular matrix

## Abstract

**Background:**

Numerous potential therapeutic alternatives for intervertebral disc degeneration (IDD) have been investigated, the most promising of which are based on biological variables such as microRNAs (miRNAs). Therefore, we verified the hypothesis that miRNAs modulate IDD by affecting the FBXO21-ERK signalling pathway.

**Methods:**

Microarray and quantitative real-time polymerase chain reaction (RT–qPCR) tests were used to examine the expression profiles of miRNAs in nucleus pulposus (NP) cells between patients with IDD and controls. Western blotting and luciferase reporter assays were used to identify the miRNA targets.

**Results:**

Microarray and RT–qPCR assays confirmed that the expression level of miR-217 was significantly decreased in degenerative NP cells. CpG islands were predicted in the miR-217 promoter region. The IDD group had considerably higher methylation than the control group. Gain- and loss-of-function experiments revealed that miR-217 mimics inhibited apoptosis and extracellular matrix (ECM) breakdown in NP cells. Bioinformatic analyses and luciferase assays were used to determine the connection between miR-217 and FBXO21. In vitro tests revealed that miR-217 mimics inhibited the expression of FBXO21, pERK, MMP13, and ADAMTS5 proteins, successfully protecting the ECM from degradation. Additionally, in vivo investigation using the IDD mouse model demonstrated that the miR-217 agonist may sufficiently promote NP cell proliferation, decrease apoptosis, promote ECM synthesis, and suppress the expression of matrix-degrading enzymes in NP cells.

**Conclusions:**

Overexpression of miR-217 inhibits IDD via FBXO21/ERK regulation.

**Trial registration:**

This study was performed in strict accordance with the NIH guidelines for the care and use of laboratory animals (NIH Publication No. 85-23 Rev. 1985) and was approved by the human research ethics committee of Wuhan University Renmin Hospital (Approval No. RMHREC-D-2020-391), and written informed consent was obtained from each participant.

**Supplementary Information:**

The online version contains supplementary material available at 10.1186/s13075-022-02949-w.

## Introduction

Intervertebral disc degeneration (IDD) is a degenerative condition that causes the intervertebral discs (IVDs), the connective tissue between the vertebrae that is critical for spinal kinematics, to degrade. Anatomically, the gelatinous nucleus pulposus (NP) of the intervertebral disc is surrounded by annulus fibrosis (AF). Degenerative processes occur at the tissue, cellular, and molecular levels, causing significant changes in the disc’s morphology and physiology and ultimately impairing its ability to withstand compressive stresses [1]. IDD is a complex disease whose aetiology is still unknown.

Numerous pathological alterations in the IVD are associated with disc degeneration, the most prominent of which are degradation of the extracellular matrix, inflammation, and cell death (apoptosis) [2]. The extracellular matrix (ECM) is critical for the mechanical performance of the IVD, and two major components are required for its integrity: the type I and type II collagen network, which provides tensile strength [3], and water-binding proteoglycans such as aggrecan [4]. However, metabolic dysregulation of NP cells decreases the ability of these cells to synthesize these ECM components while increasing their secretion of ECM catabolic enzymes such as matrix metalloproteinases (MMPs) and disintegrin and metalloproteinases with thrombospondin motifs (ADAMTS). MMP13, also known as collagenase-3, is capable of degrading collagens, while ADAMTS5 is capable of degrading aggrecan. Thus, MMP13, ADAMTS5, Collagen II, and Aggrecan are acknowledged as important biomarkers for IDD, and their functions have been demonstrated in several studies [5, 6].

Many therapeutic alternatives are being investigated and evaluated, the most promising of which are cell treatments, endogenous repair procedures based on the activation of IVD reparative cells, and treatments based on biological factors such as microRNAs (miRNAs) [7]. miRNAs, a class of short noncoding RNAs, act as essential regulators of gene expression, inhibiting translation by binding to the 3′-untranslated region (3′-UTR) of target messenger RNA molecules, frequently resulting in their degradation.

In this investigation, we conducted a complete miRNA screen in human NP tissue. miRNA expression profiling revealed a significant decrease in miR-217 expression in the NP of IDD tissues compared to control NP tissue. Additionally, miR-217 expression was negatively associated with the Pfirrmann grade of IDD. Our functional analyses demonstrated that the downregulation of miR-217 triggered IDD by promoting NP cell death via the F-box only protein 21 (FBXO21)/extracellular regulated protein kinases (ERK) pathway. Our in vivo study examining the probable consequences of intradiscal miR-217 agonist injection in mouse models demonstrated an NP-protective effect. Thus, our data establish for the first time that miR-217 may be a viable target for the prevention of IDD.

## Materials and methods

### Patient specimens

NP samples were collected from 76 patients with degenerative disc degeneration who underwent discectomy (mean age 56.8 ± 4.3 years). Surgery is indicated when conservative therapy fails and progressive neurological impairments develop, such as progressive motor weakness or cauda equina syndrome. Patients with lumbar spine stenosis, ankylosing spondylitis, isthmus or degenerative spondylolisthesis, or generalized idiopathic skeletal hypertrophy were excluded from this study. A total of 78 patients were recruited to serve as age- and sex-matched controls. Control NP tissue samples were obtained from patients who underwent anterior decompressive surgery for traumatic lumbar fractures complicated with neurological disorders. These patients received a typical lumbar spine MRI scan prior to surgery. The degree of IDD was rated using the Pfirrmann classification. The research protocol was approved by our hospital’s Ethics Committee, and each participant provided written informed permission.

### Primary nucleus pulposus cell culture and transfection

The human NP tissues were washed three times with phosphate-buffered saline (PBS; Gibco, Grand Island, NY), minced into small fragments, digested with 0.25% (w/v) trypsin and 0.2% (w/v) type collagenase (Gibco), and then placed in PBS for approximately 3 h at 37 °C in a gyratory shaker. The cells were filtered using a 70-μm mesh filter (BD, Franklin Lakes, NJ, USA). Primary NP cells were grown in 100-mm culture dishes with growth medium (Dulbecco’s modified Eagle’s medium and Ham’s F-12 nutrient mixture (DMEM-F12; Gibco), 20% (v/v) foetal bovine serum (FBS; Gibco), 50 U/mL penicillin, and 50 g/mL streptomycin (Gibco). Trypsin was used to passage the cells at approximately 80% (v/v) confluence, and the cells were subcultured in a 60-mm culture dish (2.5 × 10^5^ cells/well). The subsequent studies employed cells that had been passaged no more than twice.

Prior to transfection, NP cell culture was incubated for 24 h after controlling the initial concentration to approximately 2×10^4^ cells per well. The samples were sorted into groups and transfected with either one of several miR-217 mimics (mirVana miRNA mimics, Thermo Fisher Scientific, Waltham, MA, USA), miR-217 inhibitor (mirVana miRNA inhibitors, Thermo Fisher Scientific), mimic control (Thermo Fisher Scientific), or inhibitor control (Thermo Fisher Scientific). The transfection relied on Lipofectamine 3000 (Invitrogen, Carlsbad, CA, USA). MiR-217 mimics or inhibitors were labelled with Cy3 using the Silencer® siRNA Labelling Kit (#AM1632, Invitrogen).

### Microarray analyses

First, total RNA was extracted using the TRIzol technique from NP cells kept at a final concentration of 1 mg/mL. After purifying the miRNA component of the total RNA with the miRNA isolation kit (Ambion), the extracted miRNA samples were evaluated on the chip using the Agilent Company’s human miRNA chip (v.12.0). GeneSpring GX v12.1 software was used to process the data (Agilent Technologies).

### qRT–PCR

TRIzol reagent was used to extract total RNA from transfected cell lines (Takara, Japan). For reverse transcription, a one-step PrimeScript miRNA cDNA Synthesis Kit (Takara) was used. On the ABI 7300 system, we used SYBR Green Real-Time PCR Master Mix (Takara) to execute qRT–PCR and synthesize the data (ABI). Additionally, U6 snRNA was used as an internal control.

### Prediction of CpG islands and bisulfite sequencing PCR (BSP)

Promoter Inspector (http://www.genomatix.ed) was used to estimate the promoter area. CpG islands linked with the promoter were predicted using the CpG prediction algorithm. The Qiagen DNeasy Blood and Tissue Kit was used to isolate genomic DNA from NP, which was then placed in bisulfite. Then, using BSP primers, the genomic DNA was amplified and cloned into the pGEMT Easy vector (Promega, WI, USA). Then, the samples were sequenced, and the data were examined using a BIQ analyser.

### Luciferase assay

The 3′UTR of the FBXO21 gene fragment, containing potential binding sites of miR-217, was amplified by PCR. Mutations were generated using the QuikChange Site-Directed Mutagenesis Kit (Stratagene, La Jolla, CA, USA). Either the wild-type or mutant FBXO21-3′UTR fragment was inserted into the psi-CHECKTM-2 vector (Promega, Madison, WI) downstream of the firefly luciferase gene with XhoI and NotI (Thermo Fisher Scientific). For the luciferase assay, cultured primary human NP cells were seeded at 3000 cells per well in a 96-well plate. Cells were cotransfected with WT- or mutant-type FBXO21 3′UTR-Luc reporter plasmid and miR-control or miR-217 using Lipofectamine PLUSTM reagent (Invitrogen). Cell lysates were harvested 48 h after transfection, and luciferase activity was assayed with the Dual-Glo Luciferase Assay system (Promega, Madison, WI, USA) according to the manufacturer’s instructions. Luciferase activity was measured using the Dual-Luciferase Reporter Assay System (Promega) after 48 h. Experiments were performed in triplicate and repeated at least three times independently.

### EdU analysis

After processing the cells according to the treatment conditions and time specified for each group, each plate was replenished with 50 μmol/L EdU (Sigma–Aldrich) medium. For 2 h, the cells were grown in a 37 °C, 5% CO_2_ incubator and then rinsed twice with PBS. The cells were fixed with 4% paraformaldehyde, 0.2% glycine was added, and the cells were rinsed with PBS for 5 min. The membranes were ruptured for 10 min with 0.5% Triton-100 and rinsed with PBS, and each well was replenished with 100 μL of Apollo staining reaction solution. The plate was incubated at room temperature in the dark for 30 min on a shaker and then rinsed with 0.5% Triton-100 and 100 μL methanol and PBS. The cells were stained with DAPI for 20 min before being washed with PBS and examined under a fluorescence microscope.

### Fluorescence in situ hybridization

Complementary locked nucleic acid (LNA) probes for miR-217 were tagged with 5′ and 3′-digoxigenin (Exiqon, Woburn, MA, USA). FISH detection was performed on NP tissue from IDD patients. After the removal of a section, the gene break probe was added dropwise. The section was then placed on the hybridization instrument for 10 min at 75 °C and then incubated overnight at 42 °C. It was removed the next day and rinsed for 5 min at room temperature and 3 min at 72 °C. It was then dried, and DAPI was added dropwise to completely cover the slide. To examine and capture the image, a fluorescence microscope (Olympus IX-81; Olympus, Tokyo, Japan) was used. The percentages of miR-217 cells in three representative high-power fields from a single sample were then analysed.

### Bioinformatics analysis

Using the DAVID bioinformatics program (https://david.ncifcrf.gov/tools.jsp), GO analysis was conducted to predict the effects of downregulated mRNAs on intervertebral discs. Cytoscape (v.3.6.1) was used to design and analyse a miRNA-hub gene network to investigate the relationship between possible target genes and DE-miRNA candidates. TargetScan (http://www.targetscan.org/), miRanda (http://www.microrna.org/), PicTar (https://pictar.mdc-berlin.de/), PITA (https://genie.weizmann.ac.il/), and RNA22 (https://cm.jefferson.edu/rna22/) were used to determine the target genes of miR-217.

### Flow cytometry (FCM)

Apoptosis was determined using FITC-Annexin V and ethidium iodide (PI, 556547, BD Biosciences). Referring to the kit instructions for the operating technique, the percentage of apoptosis based on the fluorescence intensity was calculated.

### Western blot

Protein lysates were prepared from cultured primary human NP cells using RIPA buffer supplemented with protease and phosphatase inhibitors. The protein concentration of each group of cells was determined using the BCA technique, and 20 μg/well of protein was electrophoresed at 6 to 12% SDS–PAGE. The electrophoresis-separated proteins were deposited onto PVDF membranes. After incubation with the first antibody, a second antibody against rabbit immunoglobulin G (ab99697, Abcam, Cambridge, USA) was added, incubated at room temperature for 1 h, and developed with ECL reagent (Thermo Fisher Scientific, Inc.). The grey value of each protein band was analysed using ImageJ software (National Institutes of Health) to calculate the optical density.

The primary antibody information is as follows: anti-Col II antibody (ab34712, Abcam, Cambridge, USA), anti-aggrecan antibody (ab36861, Abcam, Cambridge, USA), anti-ADAMTS5 antibody (ab41037, Abcam, Cambridge, USA), anti-MMP13 antibody (ab39012, Abcam, Cambridge, USA), anti-MMP3 antibody (ab52915, Abcam, Cambridge, USA), anti-FBXO21 antibody (A16107, ABclonal, Wuhan, China), anti-ERK antibody (ab72100, Abcam, Cambridge, USA), anti-pERK antibody (ab229912, Abcam, Cambridge, USA), and anti-GAPDH antibody (ab8245, Abcam, Cambridge, USA).

### Coimmunoprecipitation

Protease and phosphatase inhibitors were added before use. Cells were collected in lysis buffer (25 mM Tris-HCl (pH 7.5), 150 mM NaCl, 0.5% NP-40). The lysate was centrifuged and immunoprecipitated using protein agarose beads and a primary antibody. Precipitated proteins and initial whole-cell lysates were boiled in an SDS loading buffer, separated on an SDS-polyacrylamide gel, transferred to a PVDF membrane, and incubated with primary and secondary antibodies.

### Cellular immunofluorescence

After the cells were seeded on glass slides in a 6-well culture plate, they were transfected the following day, and 4% paraformaldehyde was applied to the glass slides 48 h later to fix the cells on the glass slides. This was followed by incubation at 4 °C for an additional 20 min with 0.5% Triton X-100, 30 min of blocking with normal goat serum, and finally the addition of the primary antibody. These cells were treated with FITC-labelled secondary antibody at room temperature for 1 h in the dark before being washed. The nuclei were counterstained with DAPI, washed, mounted, and viewed under a fluorescence microscope to determine their structure. Detailed information on the primary antibodies is provided below: anti-Col II antibody (ab34712, Abcam, Cambridge, USA) and anti-MMP13 antibody (ab39012, Abcam, Cambridge, USA). GAPHA anti-rabbit IgG (ab150077, Abcam, Cambridge, USA) was used as a secondary antibody.

### IDD model

An IDD model was generated in this work using AF needle puncture on mice (12-week-old, male, C57BL/6, Bar Harbour Jackson Laboratory, USA) [8, 9]. The tail disc was chosen as the IDD model because of its anatomical accessibility and low surgical morbidity [10–12]. Ketamine (100 mg/kg) was chosen as the anaesthetic for mice undergoing surgery and was administered via intraperitoneal injection. After achieving general anaesthesia, the mouse was positioned on the left side, and the model surgery was performed. A short longitudinal skin incision from Co6 to Co8 was made to assist in locating the disc position for needle insertion in the tail. Next, a syringe needle was used to puncture the Co6–Co7 coccygeal discs. The syringe needle was introduced vertically into the Co6–Co7 disc and then rotated 180° in the axial direction and held for 10 s. The puncture was made parallel to the endplates through the AF into the NP using a 31-G needle, which was inserted 1.5 mm into the disc to depressurize the nucleus. The other segments were left undisturbed for contrast. All procedures were approved by the ethics committee of Wuhan University Renmin Hospital.

### Drug administration

We separated 48 male mice that had undergone IDD surgery into four groups for treatment tests (12 mice in each group). On days 3, 7, 14, and 21, a total of 10 μL of a solution containing agomiR-217/antagomiR-217 or its negative control (RiboBio, Guangzhou, China) was slowly injected into the discs multiple times. The treatments were performed as follows: (1) IDD + agomiR NC group, (2) IDD + agomiR-217 group, (3) IDD + antagomiR NC group, and (4) IDD + antagomiR-217 group. To determine the transfection efficiency of agomiR-217/antagomiR-217 or their negative controls labelled with Cy3, in vivo fluorescence imaging was performed 24 and 72 h after injection using an IVIS 200 Imaging system (Xenogen, Calliper Life Science, MA, USA). At 12 weeks after IDD surgery, the intervertebral discs of each group were collected for radiographic and histological examination.

### Histological and radiographic evaluation

Mouse intervertebral discs were fixed in 10% neutral formalin buffer for 1 week, decalcified in EDTA decalcification solution, and then sectioned. Then, using an Olympus BX51 microscope, the histological images were evaluated using haematoxylin, eosin, and saffron O-type green staining (Olympus Centre Valley, PA, USA). An improved histological grading system for intervertebral disc degeneration was created following a review of the literature on the subject [13–19]. More specifically, the cellularity and morphology of the AF, NP, and the border between the two structures were examined. The scale was based on 5 categories of degenerative changes with scores ranging from 0 points (0 in each category) for a normal disc to 15 points (3 in each category) for a severely degenerated disc. For the morphology of the NP, score 0: round shape and the NP constitutes >75% of the disc area, score 1: round shape and the NP constitutes 50–75% of the disc area, score 2: round shape and the NP constitutes 25–50% of the disc area, and score 3: round shape and the NP constitutes <25% of the disc area. For the cellularity of the NP, score 0: stellar-shaped cells with a proteoglycan matrix located at the periphery, evenly distributed; score 1: partially stellar and partially round cells, more stellar than round; score 2: mostly large, round cells, separated by dense areas of proteoglycan matrix; and score 3: large, round cells, separated by dense areas of proteoglycan matrix. For the morphology of the AF, score 0: well-organized collagen lamellae with no ruptures; score 1: inward bulging, ruptured, or serpentine fibres constitute <25% of the AF; score 2: inward bulging, ruptured, or serpentine fibres constitute 25−50% of the AF; and score 3: inward bulging, ruptured, or serpentine fibres constitute >50% of the AF. For the cellularity of the AF, score 0: fibroblasts comprise >90% of the cells, score 1: fibroblasts comprise >75–90% of the cells, score 2: intermediate, and score 3: chondrocytes comprise >75% of the cells. For the border between the NP and AF, score 0: normal, without any interruption, score 1: minimal interruption, score 2: moderate interruption, and score 3: severe interruption.

Twelve weeks after the injection, radiographs were collected. The disc height index (DHI) was used to determine the change in IVD height. The DHI was calculated by dividing the mean of the three measurements from the midline to the boundary of the middle 50% of disc width by the mean of the two neighbouring vertebral body heights. The percentage change in the DHI of punctured discs was calculated as follows: % DHI=post-punctured DHI/prepunctured DHI × 100.

### Statistical analysis

All statistical analyses were performed using SPSS 19.0 (SPSS Inc., Chicago, IL), and graphs were generated using GraphPad Prism 5 Software (Graph Pad Software, Inc., La Jolla, CA, USA). For the qRT–PCR results, we employed the Mann–Whitney *U* test. Unpaired two-tailed Student’s *t* tests were applied to evaluate the difference between two sets of data. Among multiple groups, one-way analysis of variance (ANOVA) coupled with Tukey’s post hoc test was applied to evaluate the difference. Pearson’s correlation test was employed to evaluate the associations between the expression of miR-217 and the disc degeneration grade of patients (Pfirrmann scores). A *P* value of <0.05 was considered statistically significant.

## Results

### miRNA profiles that differ between NP tissues and those from IDD patients

**Fig. 1:**
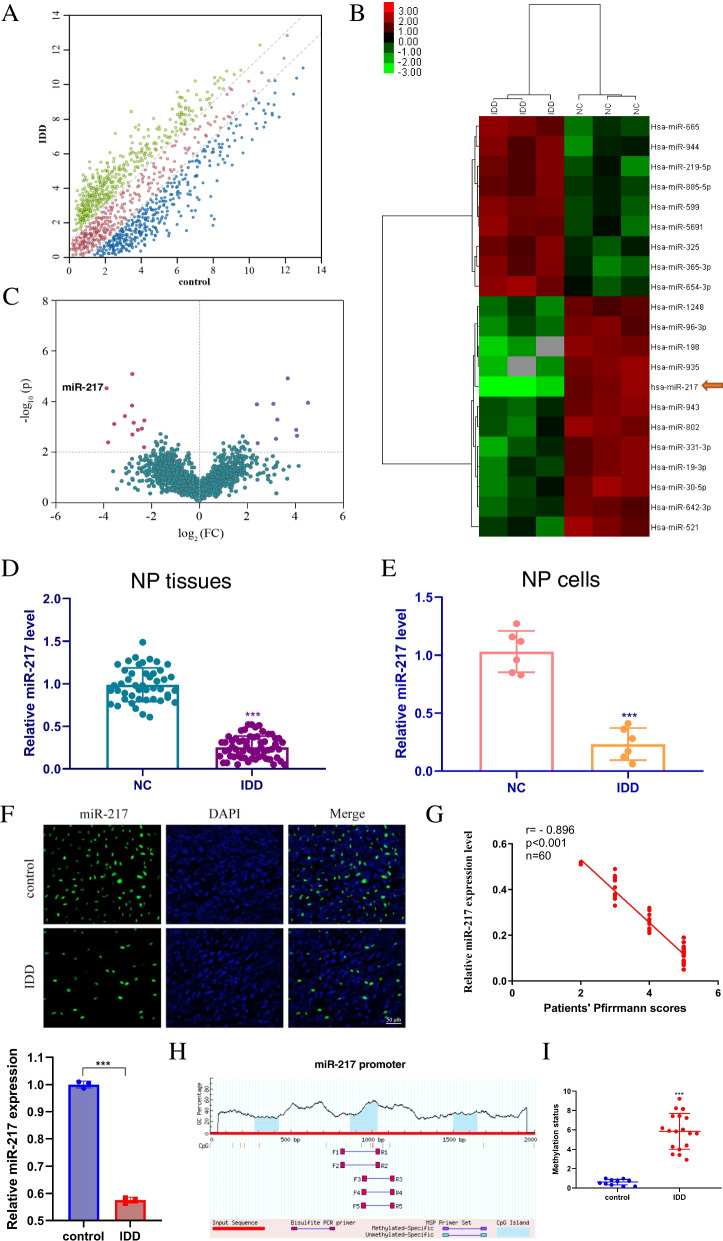
NP tissues from IDD patients showed decreased miR-217 expression. **A** Graph depicting the scatter plot of miRNA expression profiles between IDD patients and the control group (The axes represent expression fold in the IDD or control group. Genes were considered to be differentially expressed if they had an absolute log2-fold change of >2. Red dots indicate no difference between IDD and controls; green dots indicate more than a twofold increase; blue dots indicate more than a twofold decrease). **B** Heatmaps of 21 miRNAs that were found to be differentially expressed. **C** The volcano graph depicts the difference in miRNA expression levels between patients with IDD and healthy individuals in the control group. The purple dots on the right represent miRNAs that were upregulated, and the red dots on the left represent miRNAs that were downregulated. miR-217 is indicated. **D** The expression of miR-217 in human nucleus pulposus tissues was determined using qRT–PCR. **E** The expression of miR-217 in human nucleus pulposus cells was determined using qRT–PCR. **F** FISH analysis was performed on individuals with IDD as well as on the control group. Scale bar = 50 μm. **G** IDD Pfirrmann scores were shown to be negatively associated with miR-217 expression levels (*r* = −0.896, ***P < 0.001). **H** Methylation of the miR-217 promoter region. **I** Comparison of the methylation status of IDD patients (*n*=18) and controls (*n*=11). IDD, intervertebral disc degeneration; miR, microRNA; FC, fold change; DAPI, 4′,6-diamidino-2-phenylindole. ***P < 0.001

miRNAs were analysed using microarrays to comprehensively identify miRNAs that are dysregulated in IDD (Fig. 1A). These highly dysregulated miRNAs were then submitted to an unsupervised cluster analysis to distinguish IDD patients from controls, as depicted in the heatmap and volcano plots (Fig. 1B, C). Figure 1 B and C show that miR-217 was dramatically downregulated. As a result, miR-217 was chosen as the subject of future investigations. Next, miR-217 levels in human NP tissues and cells were determined using qRT–PCR assays. Figure 1 D and E show that, when compared to those in the control group, the miR-217 levels in the NP tissues and cells of the IDD group were considerably lower (*P* < 0.001). This result was verified by fluorescence in situ hybridization studies (Fig. 1F). The expression levels of miR-217 were shown to be inversely related to the Pfirrmann grade of IDD (*n*=60, *r*=−0.896, *P* < 0.001, Fig. 1G), according to our findings. CpG islands in the miR-217 promoter region were predicted to investigate the upstream mechanism of miR-217 downregulation in NP cells (Fig. 1H). The methylation status of the IDD group was found to be considerably higher than that of the control group (Fig. 1I, *P* < 0.001). It was discovered from the above findings that the amount of miR-217 in the NP tissue of IDD patients decreased while the methylation status increased.

### Overexpression or silencing of miR-217 affects the NP cell phenotype

**Fig. 2:**
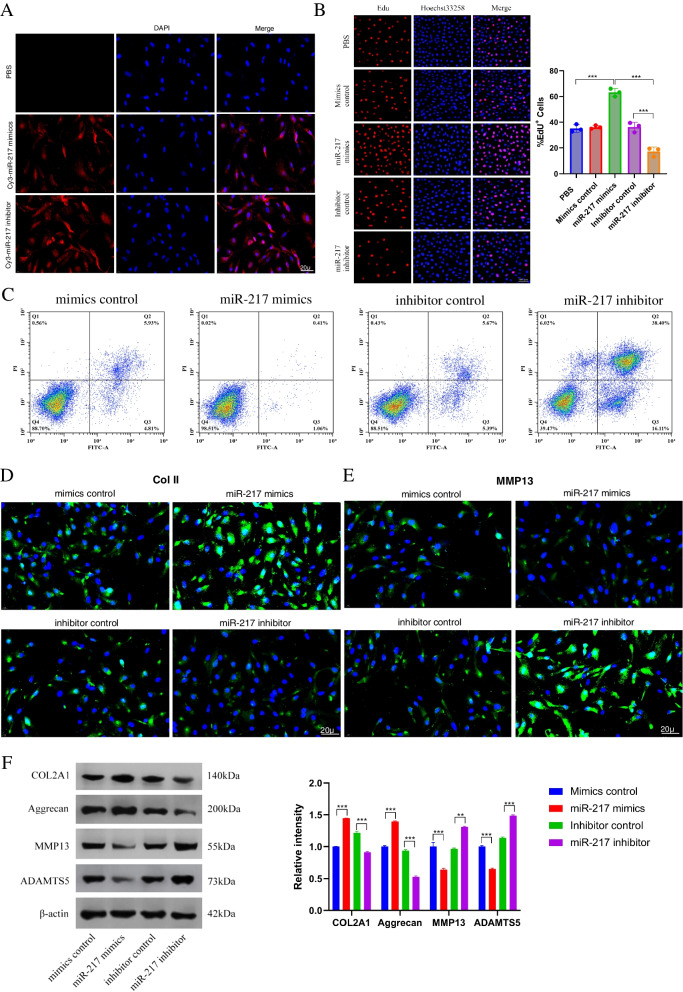
NP cell phenotypes when miR-217 was overexpressed or silenced. **A** Cy3 was employed to detect miR-217 mimics or inhibitor transfected and cultured NP cells, scale bar = 20 μm. **B** EdU assay was used to evaluate the proliferation of NP cells that were transfected with different treatments. Scale bar = 100 μm. **C** FCM was used to determine the apoptosis of NP cells. **D**, **E** Immunofluorescence was used to determine the Col II and MMP13 levels. **F** Western blotting was used to determine the levels of Col II, Aggrecan, MMP13, and ADAMT5. *n*=3. IDD, intervertebral disc degeneration; miR, microRNA; PBS, phosphate-buffered saline; DAPI, 4′,6-diamidino-2-phenylindole; FCM, flow cytometry; Col II, type II collagen; MMP, matrix metalloprotein; ADAMTS, a disintegrin-like and metalloproteinase with thrombospondin motifs; NP, nucleus pulposus; EdU, 5-ethynyl-2′-deoxyuridine

To examine the role of miR-217 in IDD, primary human NP cells were transfected with either miR-217 mimics or a miR-217 inhibitor. Using Cy3-labelled miRNA mimics or inhibitors, the transfection efficiency could be assessed (Fig. 2A). When compared to the use of a miR-217 inhibitor, the overexpression of miR-217 levels in the mimics group increased the proliferation of NP cells, according to the EdU data (Fig. 2B). Increased expression of miR-217 in NP cells was found to inhibit apoptosis in these cells (Fig. 2C). Using gain- and loss-of-function studies, we were able to further investigate the role of miR-217 levels in ECM production and the activity of matrix-degrading enzymes. The results of the immunofluorescence experiments revealed that the levels of Col II increased in primary human NP cells that had been transfected with miR-217 mimics (Fig. 2D, E). Using Western blotting, we confirmed the protein expression of MMP13, ADAMTS5, Collagen II, and Aggrecan (Fig. 2F). Overall, our research indicates that overexpression of miR-217 promotes matrix synthesis and proliferation of NP cells.

### Identification of FBXO21 as a target gene of miR-217

**Fig. 3:**
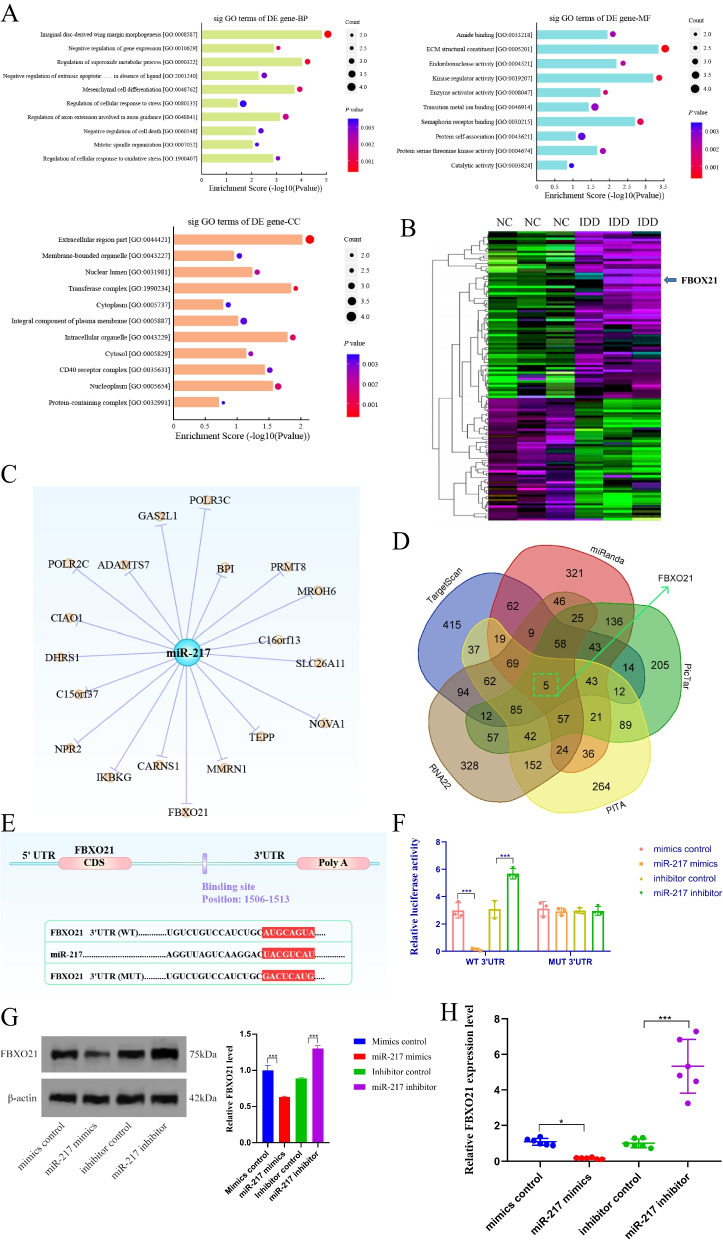
FBXO21 is directly targeted by miR-217 and is a regulator of IDD. **A** Gene ontology analysis showing the highest enrichment scores of the downregulated GO terms, including those involved in imaginal disc-derived wing margin morphogenesis, ECM structural constituent, and extracellular region. **B** Heatmap of all differentially expressed mRNAs between the sample with IDD and the paired controls. **C** The target of miR-217 was verified by Cytoscape. **D** Venn diagram displaying miR-217, computationally predicted to target FBXO21 by different algorithms. **E** The mRNA 3′UTR of FBXO21 and the putative miR-217 binding site sequence have high sequence conservation and complementarity with miR-217. **F** The wild- or mutant-type FBXO21 3′UTR reporter plasmid was either cotransfected with miR-217 mimics or inhibitor into cultured NP cells. As the 3′-UTR of wild-type FBXO21 contains miR-217 binding sites, the translation of firefly luciferase was inhibited in the miR-217 mimics group. Thus, by measuring the luciferase activity, it can be determined FBXO21 is the target gene of the miR-217. **G**, **H** The expression level of FBXO21 was evaluated by western blotting and qRT–PCR. *n* = 3. IDD, intervertebral disc degeneration; miR, microRNA; NP, nucleus pulposus; GO, gene ontology; mut, mutant; WT, wild type; FBXO21, F-box only protein 21. ***P < 0.001

We performed a Gene Ontology (GO) analysis on the dysregulated mRNAs and discovered that the GO term of the downregulated genes in the biological process that had the highest *P* value among molecular functions and cellular components was interrelated with imaginal disc-derived wing margin development (GO: 0008587), ECM structural constituent (GO: 0005201), and extracellular region part (GO: 0044421) (Fig. 3A). A heatmap was developed to identify the mRNAs that were dysregulated in IDD (Fig. 3B). In addition, using Cytoscape software, a map of the miRNA–mRNA network was created (Fig. 3C). The predicted genes were assembled into a Venn diagram to further investigate the potential targets of miR-217 (Fig. 3D). The findings indicated that miR-217 specifically targeted the FBXO21 gene (Fig. 3E). Luciferase reporter gene analysis was employed to test the association between FBXO21 and miR-217. As a result, the relative luciferase reporter activity of wild-type (WT) cells cotransfected with miR-217 mimics in primary human NP cells was shown to be significantly lower than that transfected with the mimic control (Fig. 3F, *P* < 0.001). Further confirmation of this conclusion was obtained by testing the proteins involved. The findings of the Western blot and qRT–PCR experiments indicated that the expression level of FBXO21 in the miR-217 mimic group decreased significantly (Fig. 3G, H). The results presented above demonstrated that miR-217 specifically targets the FBXO21 gene.

### MiR-217 regulates IDD via the FBXO21/ERK axis

**Fig. 4:**
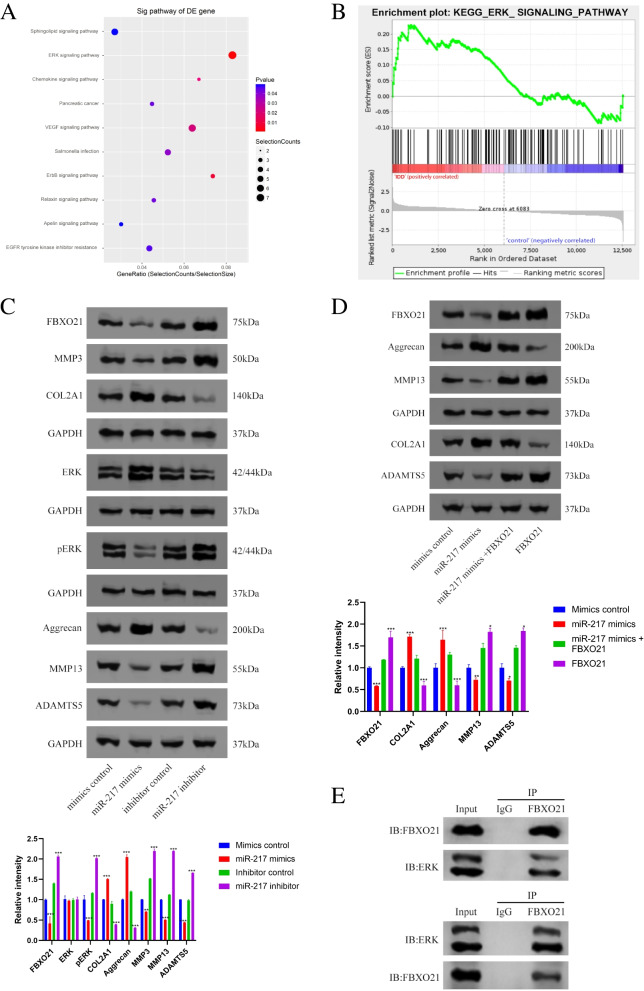
MiR-217 is involved in IDD via the FBXO21/ERK axis. **A**, **B** The Kyoto Encyclopedia of Genes and Genomes (KEGG) pathway and gene set enrichment analyses (GSEA) identified the ERK signalling pathway as the most significantly enriched pathway in intervertebral disc degeneration. **C** Western blot analysis revealed that miR-217 overexpression or silencing affected the expression patterns of FBXO21, ERK, pERK, Col II, Aggrecan, MMP3, MMP13, and ADAMT5. **D** Western blot analysis was used to determine the expression levels of FBXO21, Col II, Aggrecan, MMP13, and ADAMT5. Overexpression of FBXO21 effectively reversed the effects of miR-217 overexpression. **E** Coimmunoprecipitation revealed high-affinity physical interactions between FBXO21 and ERK. IDD, intervertebral disc degeneration; miR, microRNA; Col II, type II collagen; MMP, matrix metalloprotein; ADAMTS, a disintegrin-like and metalloproteinase with thrombospondin motifs; KEGG, Kyoto Encyclopedia of Genes and Genomes; FBXO21, F-box only protein 21; ERK, extracellular regulated protein kinases

As shown in Fig. 4 A and B, the Kyoto Encyclopedia of Genes and Genomes (KEGG) pathway and gene set enrichment analyses identified the ERK signalling pathway as the most significantly enriched pathway in intervertebral disc degeneration. To confirm that miR-217 regulates IDD through the FBXO21/ERK axis pathway, we transfected cultured primary human NP cells with miR-217 mimics, miR-217 inhibitor, or its negative controls. Western blot results demonstrated that in NP cells, the protein levels of FBXO21, p-ERK, MMP3, MMP13, and ADAMTS5 in the miR-217 mimic group declined, while in NP cells transfected with the miR-217 inhibitor, FBXO21, p-ERK, MMP3, MMP13, and ADAMTS5 protein levels increased (Fig. 4C). Moreover, a rescue experiment was carried out by overexpressing miR-217 first and then overexpressing both miR-217 and FBXO21, which indicated that FBXO21 overexpression could adequately counteract the effects of miR-217 overexpression (Fig. 4D). Importantly, a co-IP assay was performed to confirm the physical interaction between FBXO21 and ERK in degenerative NP cells (Fig. 4E). The above experimental results showed that miR-217 regulates IDD through the FBXO21/ERK axis pathway.

### Increased expression of miR-217 inhibits IDD development

**Fig. 5:**
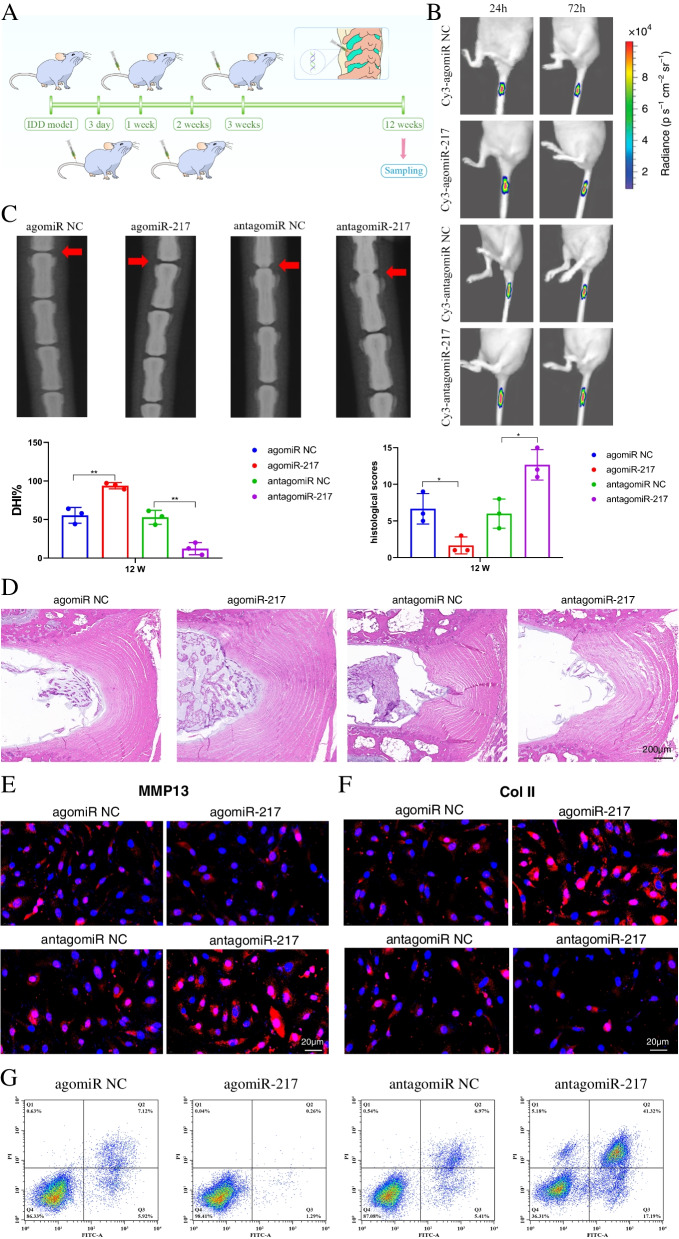
MiR-217 upregulation prevented IDD development. **A** Injections of agomiR-217, antagomiR-217, or their negative controls were performed 3, 7, 14, and 21 days after surgery. **B** Time-dependent fluorescence images of mice treated for 24 and 72 h with agomiR-217, antagomiR-217, or their negative controls labelled by Cy3. Blue to red indicates low to high intensity of the fluorescence signal. **C** X-ray examination of intervertebral disc degeneration. The DHI was tested after 12 weeks of treatment with different agents. **D** Histological findings 12 weeks after IDD surgery. In the negative control group, there was a decrease in the number of NP cells, which were replaced by cells with a more fibroblast-like phenotype. However, there was an increase in the number of NP cells in the group treated with agomiR-217 compared to the group treated with antagomiR-217 or negative controls. Moreover, the histology scores were evaluated. **E**, **F** MMP13 and Col II immunostaining in IDD models with different treatments. Scale bar = 20 μm. **G** FCM was used to evaluate apoptotic activity in the intervertebral disc; scale bar = 200 μm. IDD, intervertebral disc degeneration; NC, negative control; miR, microRNA; Col II, type II collagen; MMP, matrix metalloprotein; FCM, flow cytometry. ***P < 0.001

We used WT mice to create the IDD model and then locally administered agomiR-217/antagomiR-217 and their negative controls on days 3, 7, 14, and 21 following IDD surgery (Fig. 5A). The Cy3 labelling tests demonstrated that agomiR-217/antagomiR-217 or their negative controls had good transfection efficiency in mice (Fig. 5B). To further investigate the role of miR-217 in IDD, we carried out further procedures that included radiography and histological examination. The findings in Fig. 5 C and D demonstrated that, compared to the control group, the local administration of agomiR-217 greatly protected the IVD structure, indicating that miR-217 overexpression had a protective function on the surgically generated IDD model. The level of MMP13 was considerably reduced in groups treated with agomiR-217, while the level of Col II was significantly elevated. AntagomiR-217 had the reverse impact on MMP13 and Col II (Fig. 5E, F). Apoptosis in NP cells was examined after different treatments and was considerably reduced in mice treated with agomiR-217 (Fig. 5G).

## Discussion

It has been proven that a wide variety of microRNAs (miRNAs) are important posttranscriptional regulators with important roles in the development of human disorders. Several studies have investigated their potential impacts on IDD, and the results have been mixed [20]. In this study, we explored the involvement of miR-217 in the development of IDD. According to the findings of the current study, the expression level of miR-217 was significantly lowered in IDD tissues and was found to be negatively associated with the severity of disc degeneration. As a result of these observations, it is hypothesized that decreased NP cell proliferation caused by miR-217 downregulation may be one of the potential causes of IDD development.

To better understand the function of miR-217 level in NP cells, the effect of miR-217 level and silencing miR-217 on the phenotype of NP cells were investigated by manipulating the level of miR-217. The results showed that miR-217 has a positive effect on the phenotype of NP cells and that overexpression of miR-217 is related to increased proliferation of NP cells, inhibition of apoptosis, increased ECM formation, and the inhibition of matrix-degrading enzymes. These phenotypic alterations are the key biological process of IDD. The increase in ECM formation is manifested by an increase in the levels of Col II and aggrecan and a decrease in the expression of ADAMTS5 and MMP13 [21, 22]. Thus, miR-217 is involved in the pathogenesis of IDD.

It is well known that microRNAs (miRNAs) can participate in gene expression networks by direct interaction with the 3′ UTR of the targeted mRNAs. As a result, possible direct gene targets of miR-217 were identified in databases that were previously shown to be associated with the pathophysiology of IDD. After a thorough search and comparison using sequence complementarity algorithms, FBXO21 was chosen as the most promising candidate. Luciferase activity was dramatically reduced in NP cells transfected with miR-217 mimics when compared with cells transfected with the mimic control. This was observed in both NP cells and cells transfected with the miR-217 control. The enzymatic activity of cells transfected with the miR-217 inhibitor rose dramatically compared to that of cells transfected with the inhibitor control. Following these findings, it was discovered that miR-217 may directly interact with the 3′ UTR of FBXO21 transcripts, thereby reducing the translational activity of the chimeric transcripts. These findings were corroborated by qRT–PCR and Western blot analyses, which demonstrated that FBXO21 expression was decreased in NP cells transfected with miR-217 mimics. According to a review of prior studies, there was no mention of miR-217 having an inhibitory effect on FBXO21 in IDD. These findings revealed that FBXO21 functions as a mediator of downstream signal transduction from miR-217 in the pathogenetic mechanism of IDD.

FBXO21, a member of the F-box protein family, is a subunit of the Skp1-cullin-F-box (SCF) ubiquitin E3 ligases, which are involved in phosphorylation-dependent ubiquitination degradation [23, 24]. ERK is a serine/threonine protein kinase that can be triggered by cytokines [25]. A previous study demonstrated that FBXO21/ERK is related to the occurrence of osteoarthritis, and ERK is one of the novel binding partners of FBXO21 detected in osteoarthritis [26]. Once FBXO21 binds to ERK, it induces chondrocytes to release various enzymes (MMP3, MMP13, and ADAMTS5), leading to the degradation of type II collagen and aggrecan. In this study, we further demonstrated that miR-217 affects the alterations in the NP phenotype in IDD by acting on the FBXO21/ERK signalling pathway. In our work, the ERK signalling pathway was found to be markedly enriched in NP cells, as determined by KEGG pathway analysis. Furthermore, we discovered that miR-217 mimics greatly lowered the phosphorylation of ERK, as well as the expression of MMP3, MMP13, and ADAMTS5. However, this effect of miR-217 mimics was abrogated by FBXO21 treatment. Therefore, through functional studies performed in vitro, we verified that miR-217 suppresses the FBXO21/ERK signalling pathway in NP cells.

The in vivo analysis using an inducible IDD animal model further revealed the important clinical significance of miR-217 and its downstream pathways. As expected, in terms of protecting the phenotype of NP cells, overexpression of miR-217 was found to be effective in alleviating the symptoms of IDD in mouse models. This is not the first time that microRNAs (miRNAs) have been shown to be beneficial in the treatment of IDD. However, it should be noted that the amount of miRNAs produced may be influenced by the methylation of the miRNA promoter region, which may have an impact on specific phenotypic characteristics [27, 28]. In this study, strong CpG islands were discovered in miR-217-associated promoters based on the prediction of CpG islands in the promoter, which was confirmed by the results. Consequently, we believe that miR-217 in its host gene may be regulated by promoter methylation to function correctly. More studies are needed to confirm that hypermethylation may be a factor in the loss of miR-217 in NP cells.

## Conclusions

MiR-217 alleviates IDD by targeting FBXO21/ERK, which regulates NP cell proliferation, apoptosis, and ECM breakdown. These findings establish the groundwork for future research, as well as for a better understanding and treatment of IDD while also identifying a prospective therapeutic target for IDD treatment.

## Supplementary Information

**Additional file 1.** Full-length Western blot images.

## Data Availability

All data generated or analysed during this study are included in this published article.
