# Supplementary figures and images for: Epigenetic modification of miR-217 promotes intervertebral disc degeneration by targeting the FBXO21-ERK signalling pathway
Source: Arthritis Res Ther. 2022 Nov 28;24:261. doi: 10.1186/s13075-022-02949-w (PMC9703697; doi:10.1186/s13075-022-02949-w)

Figure 2F

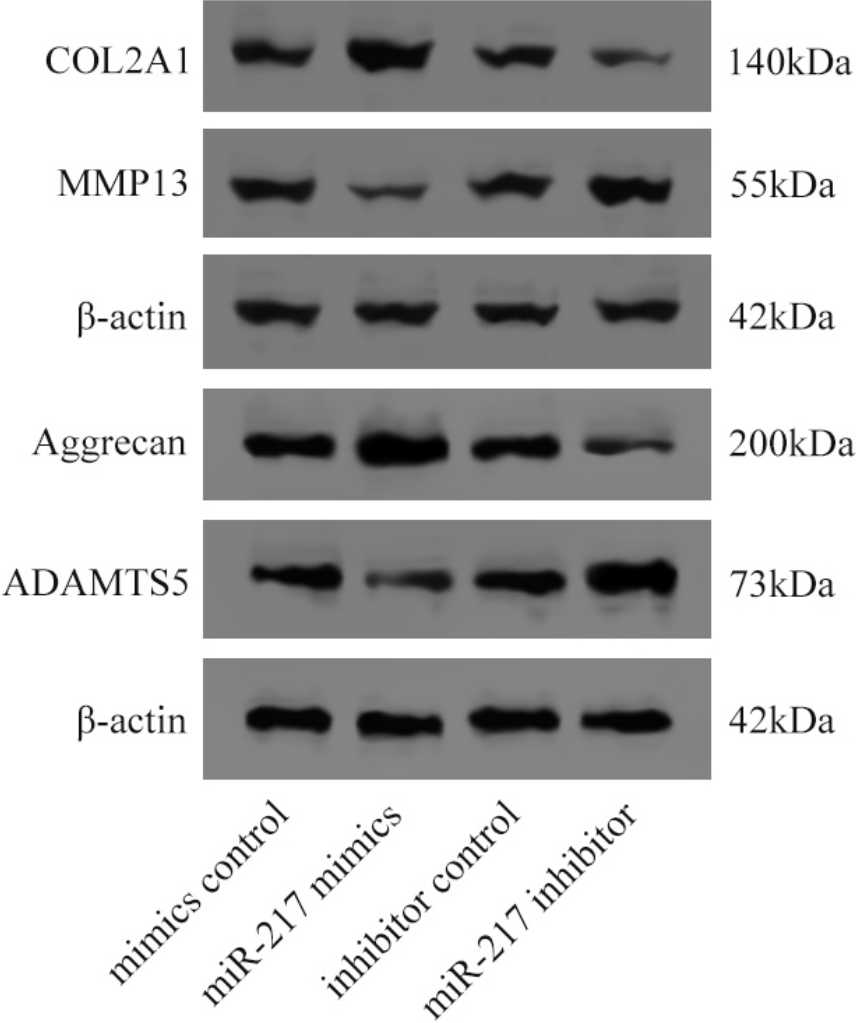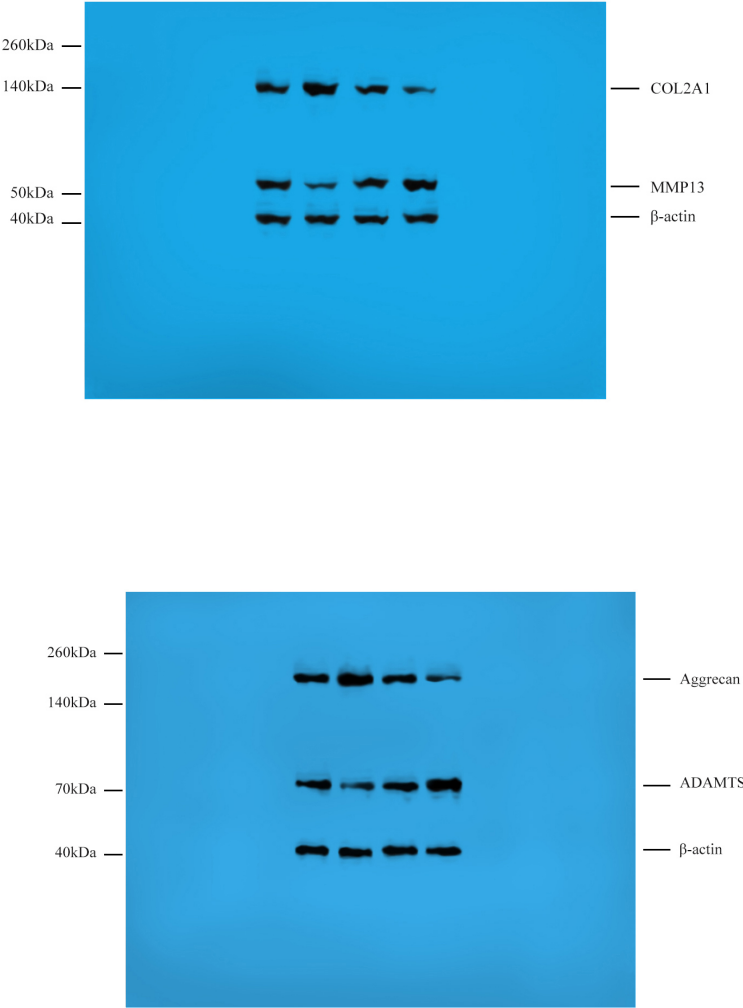

Figure 3G

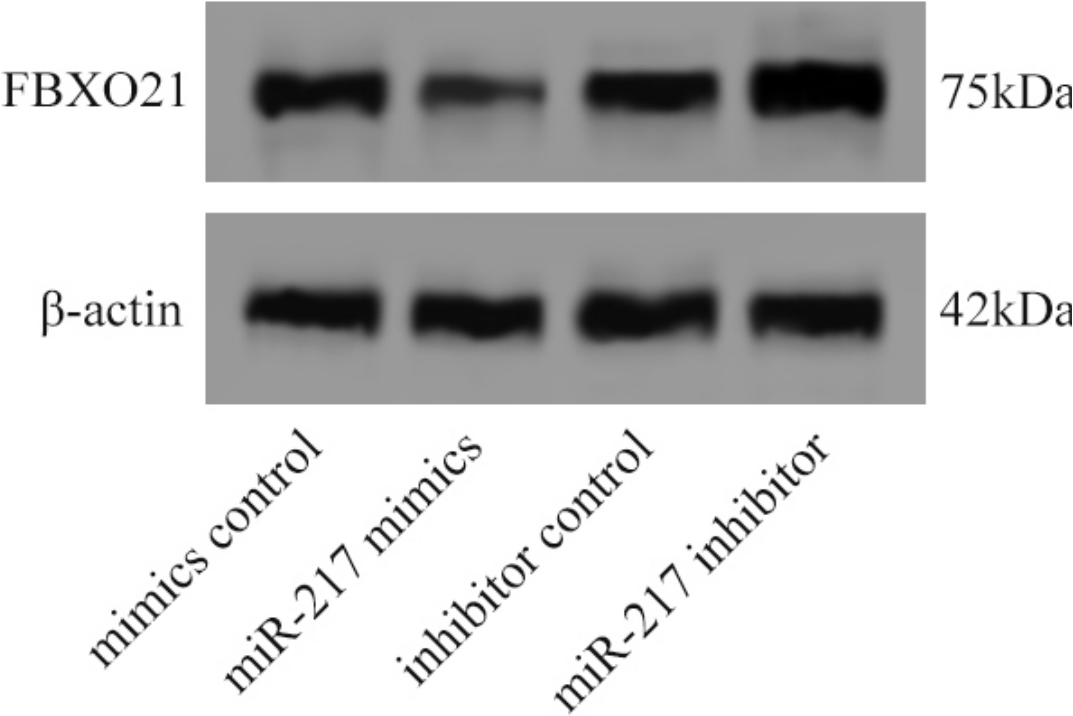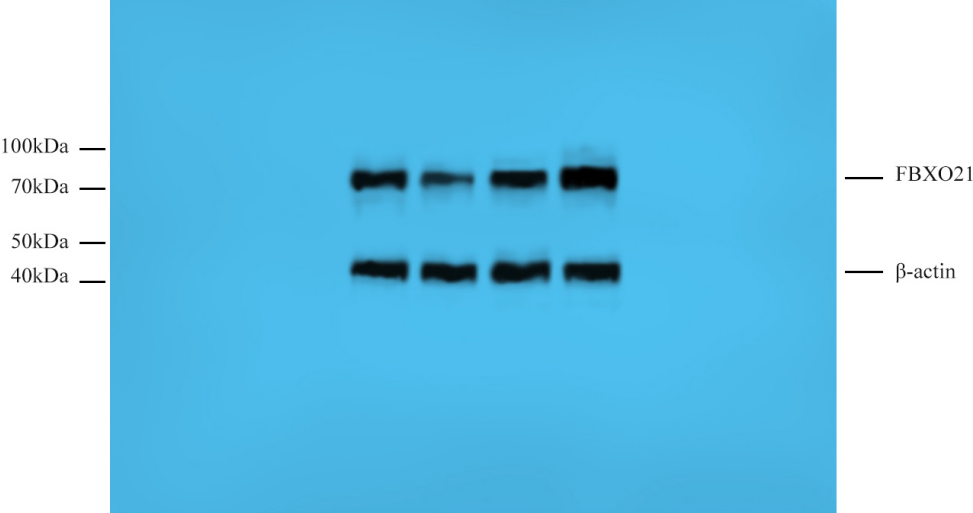

Figure 4C

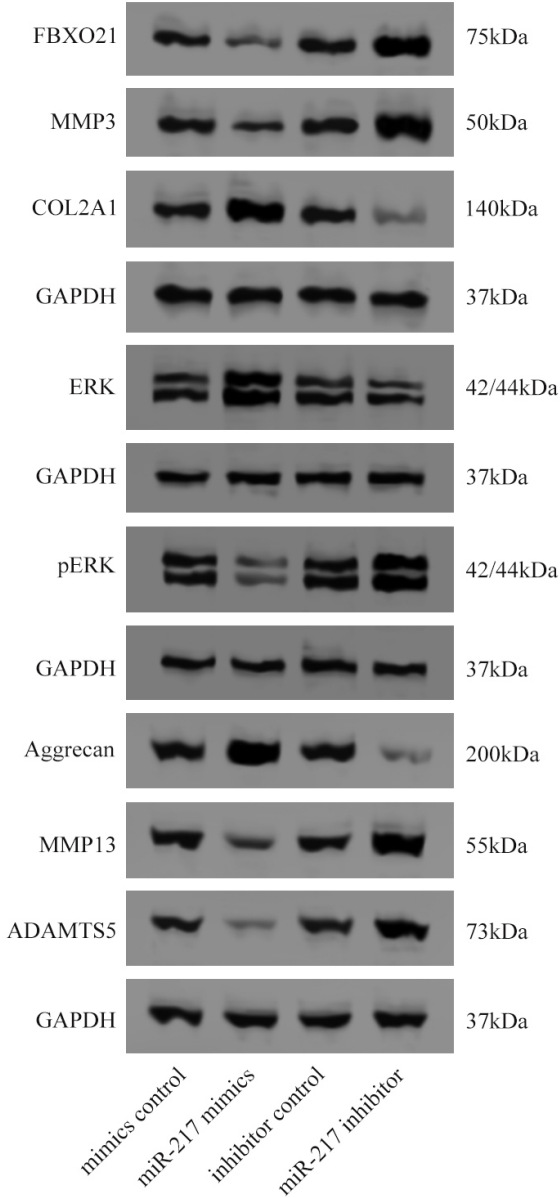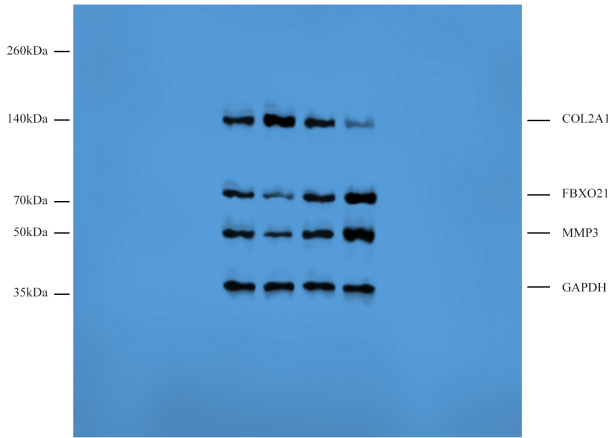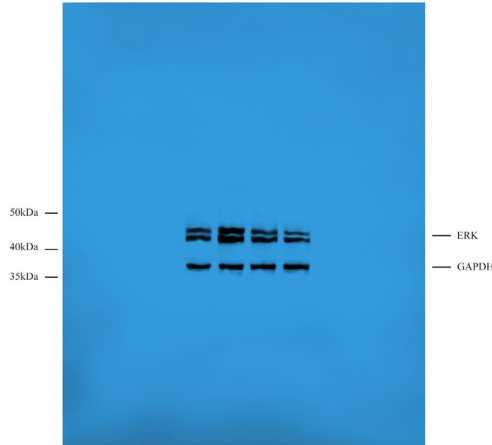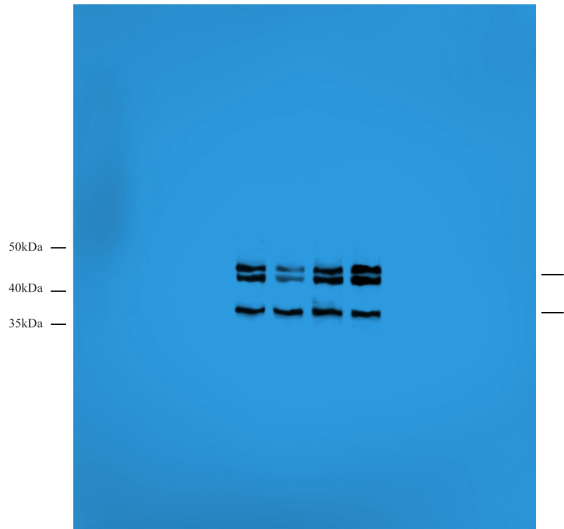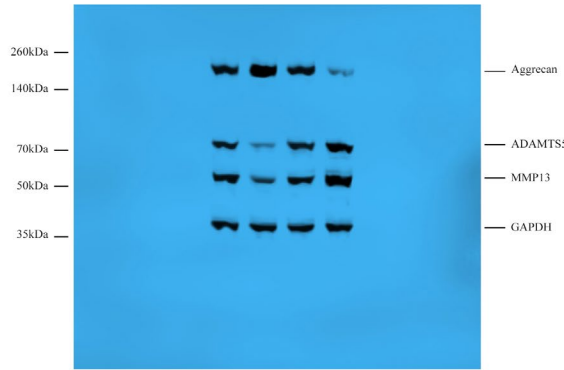

Figure 4D

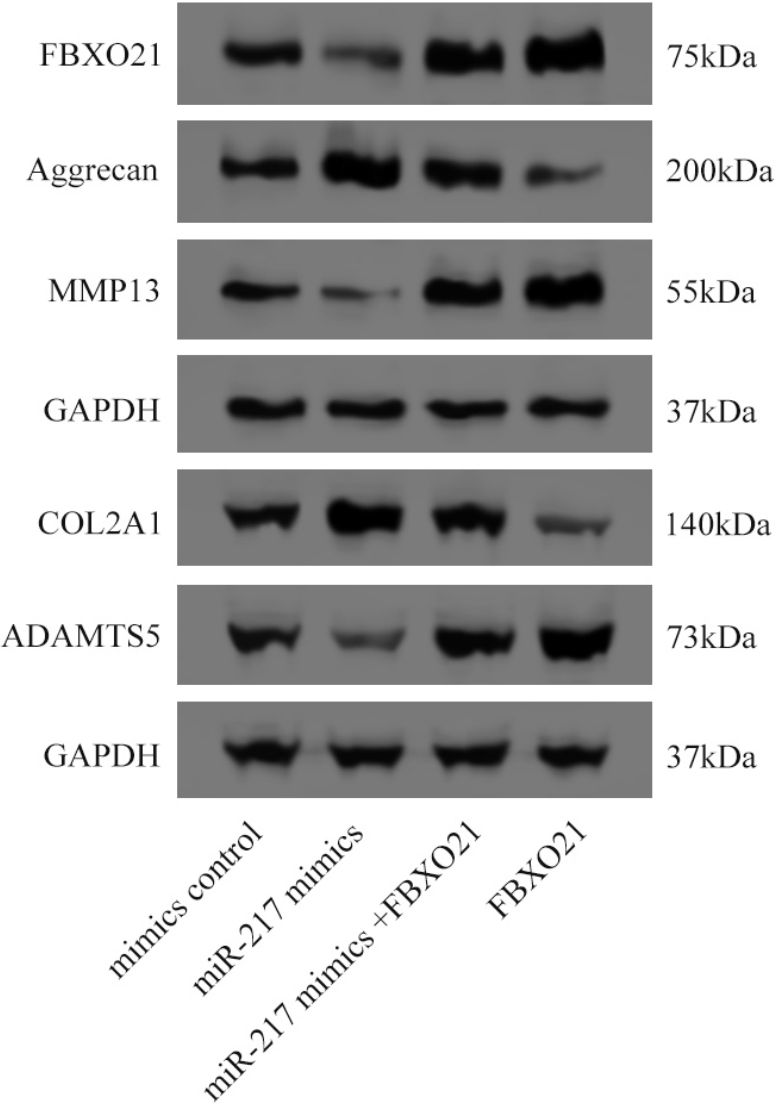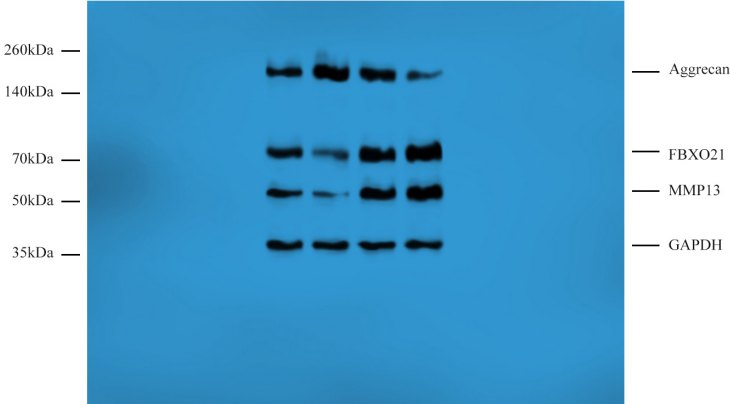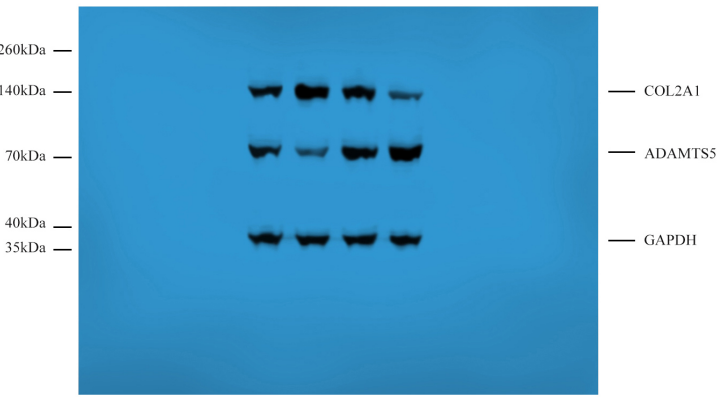

Figure 4E

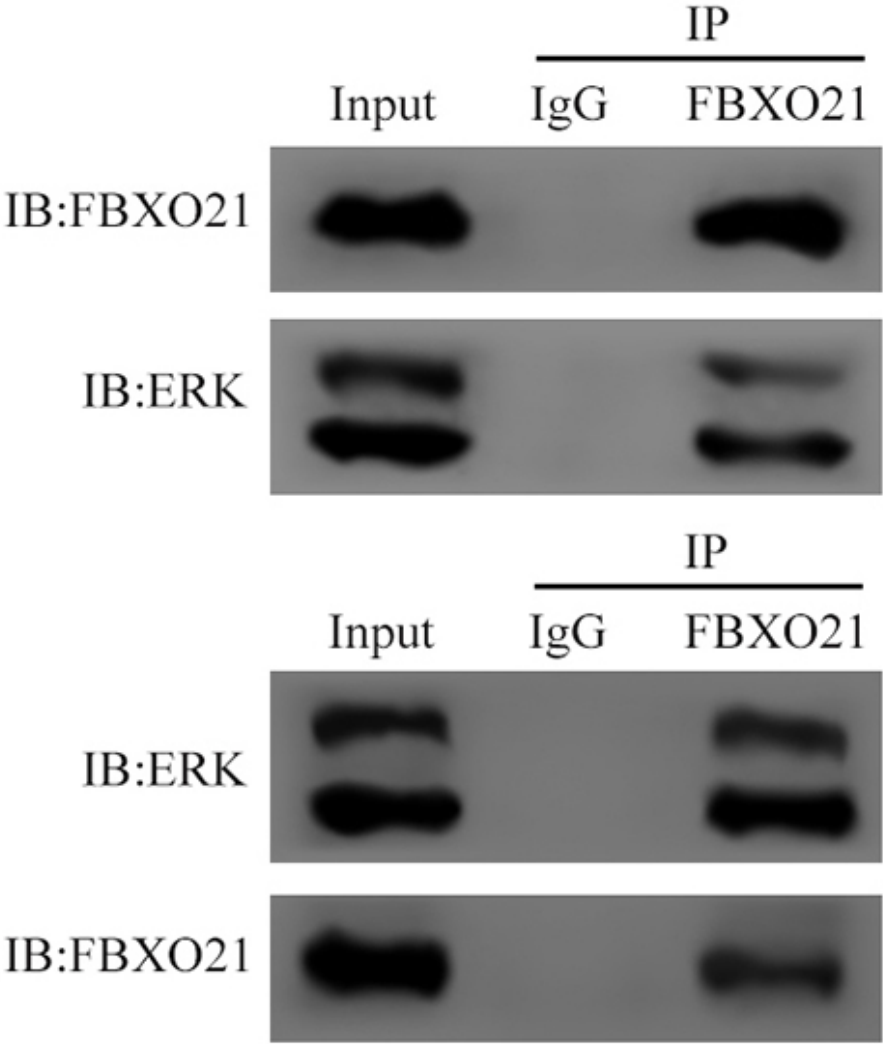

IB:FBXO21

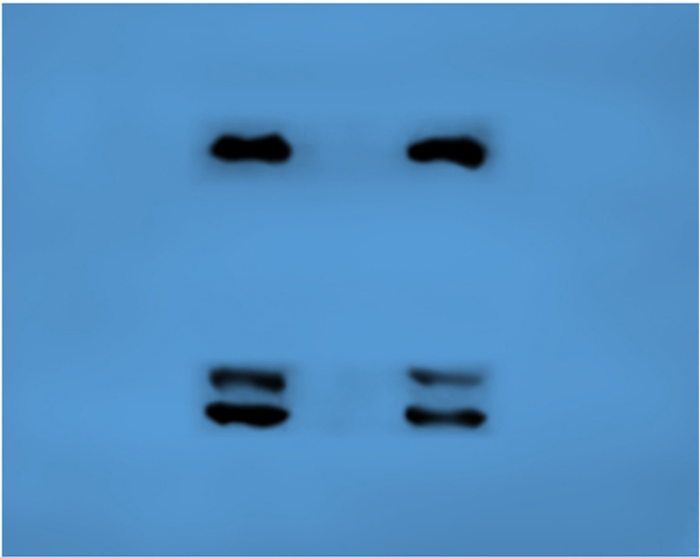

IB:ERK

IB:ERK

IB:FBXO21

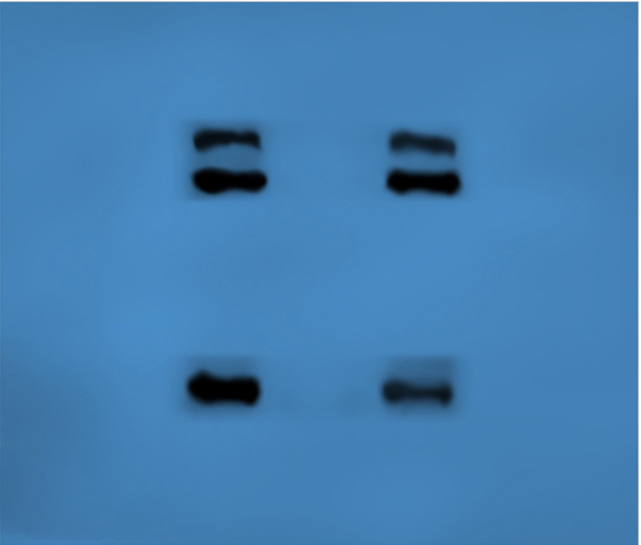

Supplement: Supplementary file 1 — Additional file 1. Full-length Western blot images. [file 13075_2022_2949_MOESM1_ESM.pdf]
